# Identification of miR-143-3p as a diagnostic biomarker in gastric cancer

**DOI:** 10.1186/s12920-023-01554-3

**Published:** 2023-06-16

**Authors:** Yeongdon Ju, Go-Eun Choi, Moon Won Lee, Myeongguk Jeong, Hyeokjin Kwon, Dong Hyeok Kim, Jungho Kim, Hyunwoo Jin, Kyung Eun Lee, Kyung-Yae Hyun, Aelee Jang

**Affiliations:** 1 Medical Science Research Center, Pusan National University, Yangsan, 50612 Republic of Korea; 2 Department of Clinical Laboratory Science, College of Health Sciences, Catholic University of Pusan, Busan, 46252 Republic of Korea; 3 Division of Gastroenterology, Pusan National University Hospital, Busan, 49241 Republic of Korea; 4 Department of Internal Medicine, Pusan National University College of Medicine, Busan, 49241 Republic of Korea; 5 Department of Clinical Laboratory Science, Dong-Eui University, Busan, 47340 Republic of Korea; 6 Department of Nursing, University of Ulsan, Ulsan, 44610 Republic of Korea

**Keywords:** Gastric cancer, miR-143-3p, Biomarker, Bioinformatics, Diagnosis

## Abstract

**Background:**

Gastric cancer (GC) is among the most common types of gastrointestinal cancers and has a high incidence and mortality around the world. To suppress the progression of GC, it is essential to develop diagnostic markers. MicroRNAs regulate GC development, but a clearer insight into their role is needed before they can be applied as a molecular markers and targets.

**Methods:**

In this study, we assessed the diagnostic value of differentially expressed microRNAs as potential diagnostic biomarkers for GC using data for 389 tissue samples from the Cancer Genome Atlas (TCGA) and 21 plasma samples from GC patients.

**Results:**

The expression of hsa-miR-143-3p (also known as hsa-miR-143) was significantly downregulated in GC according to the TCGA data and plasma samples. The 228 potential target genes of hsa-miR-143-3p were analyzed using a bioinformatics tool for miRNA target prediction. The target genes correlated with extracellular matrix organization, the cytoplasm, and identical protein binding. Furthermore, the pathway enrichment analysis of target genes showed that they were involved in pathways in cancer, the phosphoinositide 3-kinase (PI3K)–protein kinase B (Akt) signaling pathway, and proteoglycans in cancer. The hub genes in the protein–protein interaction (PPI) network, were matrix metallopeptidase 2 (MMP2), CD44 molecule (CD44), and SMAD family member 3 (SMAD3).

**Conclusions:**

This study suggests that hsa-miR-143-3p may be used as a diagnostic marker for GC, contributing via the pathways involved in the development of GC.

**Supplementary Information:**

The online version contains supplementary material available at 10.1186/s12920-023-01554-3.

## Background

Gastric cancer (GC) is the most common cancer and the leading cause of cancer deaths globally [1, 7]. GC imposes a significant health burden worldwide due to the low survival rates of patients with advanced disease. This is because of the lack of effective biomarkers for early detection and few effective therapies for the patients in advanced stage [2]. To date, the main etiologies of GC have been reported to be genetic and environmental factors, with *Helicobacter pylori* infection being identified as the most prominent [2–4]. Recently, many researchers have concentrated on investigating the pathophysiologic mechanisms of GC [5]. The molecular mechanisms and prognostic values of stromal immune signature are implicated in the progression of GC. However, the molecular mechanisms of GC development are not fully uncovered [6, 7]. The molecular markers and targets for the cancer are still in their infancy and the biomarker-based approaches with hypothetical benefit are needed to achieve the diagnosis of GC [8].

MicroRNAs (miRNAs) are short, endogenous RNA molecules that are 19–24 ribonucleotides in length [9]. The dysregulation of miRNAs has an important influence on the progression of human cancer, and the expression of miRNAs may influence the tumorigenesis, progression, invasion, and metastasis of GC [9–12]. miRNAs play a role as diagnosing several cancers such as glioblastoma, breast cancer, and gastric cancer [13–15]. The lack of biomarkers for the identification of GC is one of the greatest challenges in the diagnostic field, the use of miRNAs as biomarkers for GC could be advantageous for the development of personalized medicine [16].

Here, we investigated differentially expressed (DE) miRNAs and their diagnostic value in GC. First, we evaluated the diagnostic usefulness of DE miRNAs as potential biomarkers for GC tissues using Cancer Genome Atlas (TCGA) data. Subsequently, we used high-throughput RNA sequencing to obtain a miRNA expression profile for plasma from GC patients, in which we identified DE miRNAs. Finally, multiple bioinformatic analyses of the GC data from the miRNA expression profile were applied to evaluate the underlying mechanisms regarding potential target mRNAs. Our study provides valuable information on a potential biomarker for the diagnosis of GC.

## Methods

### Data extraction from the Cancer Genome Atlas (TCGA): gastric cancer

The sequencing data for miRNAs, clinical information for GC tumors, and normal control tissue data were downloaded from the TCGA data portal (http://firebrowse.org/). TCGA is one of the major cancer genomics datasets and is useful for advancing our biomolecular understanding [17, 18]. miRNA sequencing was carried out using an Illumina HiSeq platform. The clinical diagnostic values were verified using miRNA expression data (reads per million mapped) for 389 GC samples and 41 non-tumor tissues. The analysis of these miRNA expression data detected a total of 1046 miRNAs in the tissue samples. Table 1 presents basic information on the study cohort including the genders, ages, pathological stages, and tumor-node-metastasis (TNM) classifications.

**Table 1:** Clinical characteristics of the Cancer Genome Atlas data

Cohorts	Analysis set, *n* (%)	Validation set, *n* (%)
Characteristic	Gastric cancer (*n* = 272)	Gastric cancer (*n* = 117)
*Gender*, *n* (%)		
Male	168 (61.8)	88 (75.2)
Female	104 (38.2)	29 (24.8)
*Age (years)*		
Mean ± SD	65.33 ± 10.50	65.08 ± 10.64
NA	5	–
*Pathologic stage*, *n* (%)		
Stage I	35 (12.9)	16 (13.7)
Stage II	102 (37.5)	22 (18.8)
Stage III	113 (41.5)	61 (52.1)
Stage IV	17 (6.3)	15 (12.8)
NA	5 (1.8)	3 (2.6)
*Primary tumor (T)*, *n* (%)		
T1	11 (4.0)	10 (8.6)
T2	63 (23.2)	15 (12.8)
T3	119 (43.8)	62 (53.0)
T4	79 (29.0)	30 (25.6)
*Regional lymph node (N)*, *n* (%)		
N0	95 (34.9)	26 (22.2)
N1	68 (25.0)	32 (27.4)
N2	49 (18.0)	30 (25.6)
N3	56 (20.6)	26 (22.2)
NX	3 (1.1)	3 (2.6)
NA	1 (0.4)	–
*Distant metastasis (M)*, *n* (%)		
M0	245 (90.1)	102 (87.2)
M1	13 (4.8)	10 (8.5)
MX	14 (5.1)	5 (4.3)

Data are expressed as the means ± standard deviations (SDs)

*NA* not available

### Patients and samples

GC Plasma samples were obtained from 21 GC patients at the Pusan National University Hospital. Normal healthy donors were drawn for control with the patient population according to an institutional review board approved protocol with informed consent. Twenty-one subjects with gastric cancer and seventeen without tumors were enrolled in our study. The clinicopathological types of 21 GC patients were reviewed by an oncologist. The details of their clinical features, along with their genders, ages, and TNM classifications, were obtained. Pathological types of gastric cancer patients were 13 tubular adenocarcinoma and 8 poorly cohesive carcinoma (Table 2). Whole blood samples were collected in ethylenediaminetetraacetic acid (EDTA) blood collection tubes. The plasma fraction was centrifuged at 2000×*g* for 10 min at 4 °C and stored at − 80 °C [19].

**Table 2:** Clinical characteristics of gastric cancer patients and healthy control subjects

Characteristic	Gastric cancer		Healthy control	
	RNA-Seq	qPCR	RNA-Seq	qPCR
Total	6	15	2	15
*Gender*				
Male	5	12	1	8
Female	1	3	1	7
*Age (years)*				
Mean ± SD	45.33 ± 16.67	57.67 ± 11.29	50.50 ± 2.12	49.87 ± 7.87
*Primary tumor* (T)				
T1	–	1	–	–
T2	–	–	–	–
T3	2	4	–	–
T4	4	10	–	–
*Regional lymph node (N)*				
N0	–	1	–	–
N1	2	3	–	–
N2	1	4	–	–
N3	3	7	–	–
*Distant metastasis (M)*				
M0	5	3	–	–
M1	1	4	–	–
MX	–	8	–	–
*Histological type*				
Tubular adenocarcinoma	3	10	–	–
Poorly cohesive carcinoma	3	5	–	–

Data are expressed as the means ± standard deviations (SDs)

### RNA extraction and qualification

Total RNA was isolated using the TRIzol method (Invitrogen, CA, USA) according to the manufacturer’s instructions. The concentrations of the RNA samples were determined using a NanoDrop 2000 spectrophotometer (Thermo Fisher Scientific, Waltham, MA, USA). The RNA integrity was evaluated using an RNA 6000 Pico Chip (Agilent Technologies, Amstelveen, the Netherlands), and the RNA quality was tested with an Agilent 2100 Bioanalyzer instrument (Agilent Technologies, Amstelveen, the Netherlands).

### Library preparation and high-throughput RNA sequencing

Library construction was carried out using a NEBNext Multiplex Small RNA Library Prep Kit (New England Biolabs, MA, USA) following the manufacturer’s protocols. To study the total RNA from each sample, we used 1 µg to ligate the adaptors for cDNA synthesis using a reverse transcriptase with adaptor-specific primers. After library amplification based on polymerase chain reactions (PCRs), we performed clean-up using a QIAquick PCR Purification Kit (Qiagen, Hilden, Germany) and AMPure XP beads (Beckmancoulter, Pasadena, CA, USA). The small RNA libraries were analyzed on the Agilent 2100 Bioanalyzer instrument (Agilent Technologies, Amstelveen, the Netherlands). A NextSeq500 system (Illumina, CA, USA) supporting single-end 75 bp sequencing was employed for high-throughput RNA sequencing.

### Data analysis

To obtain the BAM file, the sequence reads were mapped using the Bowtie2 software. From the alignment file (BAM file), we extracted the counts of reads mapped on mature miRNA sequences using the bedtools 2.25.0 and Bioconductor software based on the free open-source R language 3.2.2 [20]. To estimate the expression levels of miRNAs between multiple samples, read counts were used. For a comparison between samples, the subsequent data were normalized using the counts per million (CPM) and trimmed mean of m-values (TMM) methods [21].

### Quantitative real-time PCR analysis

Quantitative real-time PCR (qPCR) assays were performed using the miRCURY LNA miRNA PCR Assays (Qiagen Sciences, Germantown, MD, USA) and miRCURY LNA™ SYBR Green PCR kit (Qiagen Sciences, Germantown, MD, USA) attending to manufacturer’s instructions. The miRCURY LNA™ RT Kit (Qiagen Sciences, Germantown, MD, USA) was applied for performing reverse-transcription reaction on RNA. The miRCURY LNA™ SYBR Green PCR kit (Qiagen Sciences, Germantown, MD, USA) was applied for implement of qPCR. The cel-miR-39-3p was used as a spike-in reference. RNA expression was calculated using the 2^−ΔΔCt^ method. Thermocycler was used for this procedure with the following temperature program: 95 °C for 2 min, followed by 40 cycles of 10 s at 95 °C and 1 min at 56 °C for 50 cycles. The qPCR was run in triplicates on a QuantStudio 7 Flex Real-Time PCR system (Applied Biosystems, Foster City, CA, USA). A list of sequencing primers can be found in Table 3.

**Table 3:** Quantitative real-time PCR SYBR green assay sequences

miRbase ID	Mature miRNA sequence (5′ → 3′)	miRbase accession	GeneGlobe ID
hsa-miR-143-3p	UGAGAUGAAGCACUGUAGCUC	MIMAT0000435	YP00205992
cel-miR-39-3p	UCACCGGGUGUAAAUCAGCUUG	MIMAT0000010	YP00203952

### microRNA target prediction

Potential target mRNAs were identified using the miRNA target prediction database miRNet (https://www.mirnet.ca/). miRNet was established as a simple to use high-performance web-based tool to help with miRNA functional analysis and visual exploration [22].

### Gene ontology and KEGG enrichment

To obtain further insight into the function of predicted target genes, Gene Ontology (GO) analysis was performed to identify the enrichment results for biological processes (BP), cellular components (CC), and molecular functions (MF) using the DAVID bioinformatics resources (https://david.ncifcrf.gov/) [23]. The GO terms were interrogated with QuickGO (https://www.ebi.ac.uk/QuickGO/), a web-based tool [24]. The Kyoto Encyclopedia of Genes and Genomes (KEGG) pathway enrichment analysis of key modules was visualized using the Cytoscape 3.9.1 software. For KEGG pathway enrichment analysis, we established statistical options based on a two-sided hypergeometric test with Bonferroni step-down correction, a *p*-value < 0.001, and kappa scores ≥ 0.4 as the criteria.

### Protein–protein interaction network construction

For protein–protein interactions (PPIs), we used the Search Tool for Retrieval of Interacting Genes/Proteins (STRING) plugin in the Cytoscape 3.9.1 software with CytoHubba to construct a network. STRING (https://string-db.org/) is a knowledgebase for known and predicted protein interactions [25].

### Statistical analysis

Statistical analyses were programmed in the Statistical Product and Service Solutions (SPSS) 26.0 software (IBM, NY, USA) and GraphPad Prism 6.0 software (GraphPad Software, CA, USA). *P* < 0.05 was considered to indicate a statistically significant difference.

## Results

### Differentially expressed microRNA expression analysis of the TCGA data

**Fig. 1:**
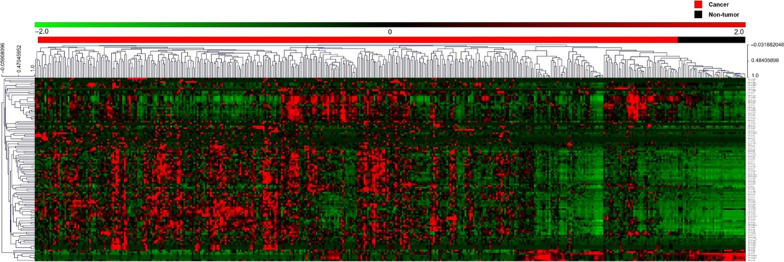
Heatmap for the identification of differentially expressed miRNAs. The two-way hierarchical clustering analysis of the 111 differentially expressed miRNAs in gastric cancer and adjacent non-tumor tissues. Red and black in the bars represent gastric cancer and non-tumor tissues, respectively. Red and green dots indicate up- and downregulation, respectively

The DE miRNA expression data of 389 GC tissues and 41 adjacent non-tumor tissues were obtained from the TCGA database. The differential expression of the 111 miRNAs between the GC and non-tumor tissues was analyzed and presented using a heatmap (Fig. 1), which was visualized using MultiExperiment Viewer (MeV) 4.9.0 (adjusted by Bonferroni correlation and *P* < 0.01 according to hierarchical clustering analysis). The 111 DE miRNAs are listed in Additional file 1: Table S1.

### Selecting 13 potential microRNAs as diagnostic biomarkers for the TCGA data

**Fig. 2:**
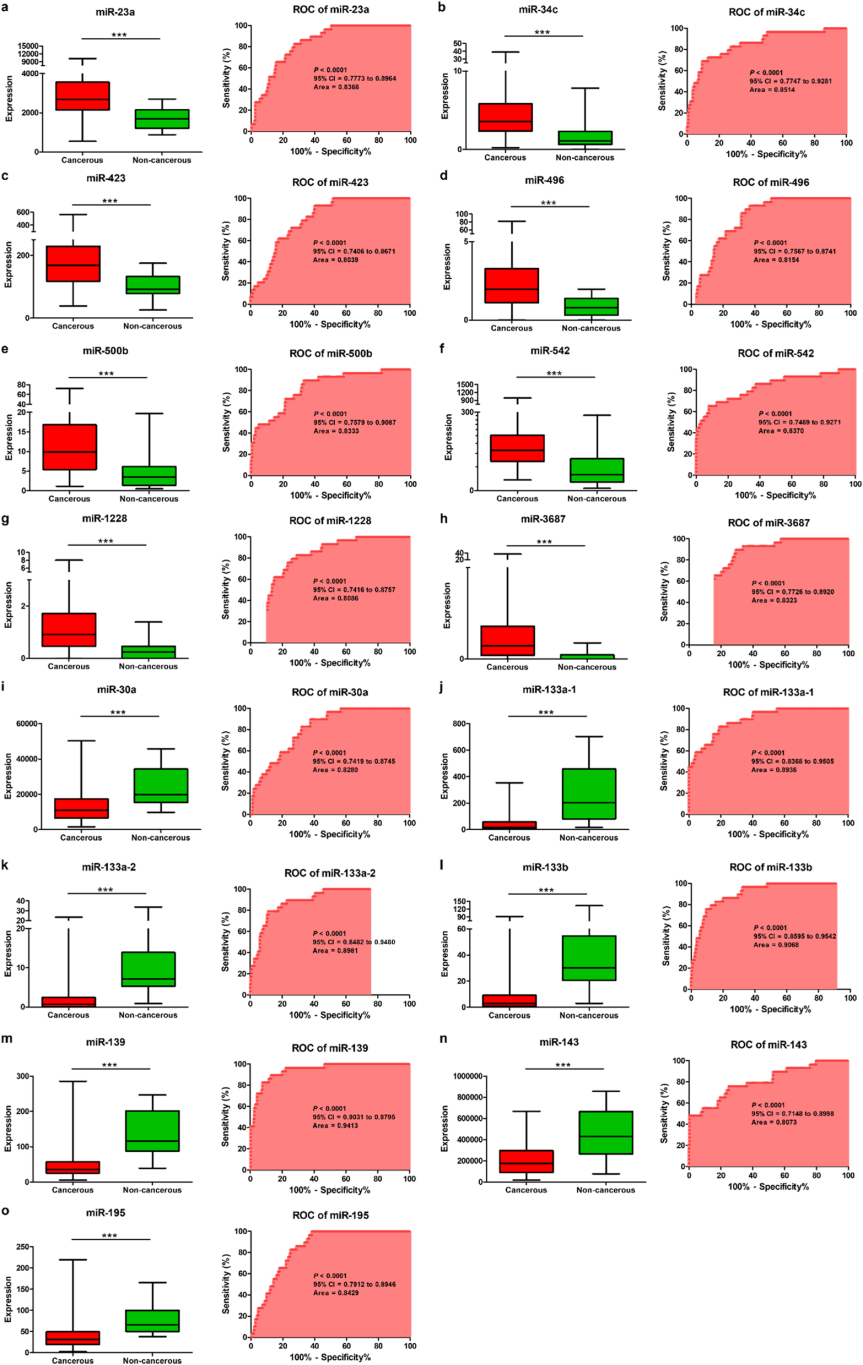
Expression levels of 15 differentially expressed miRNAs and receiver operating characteristic curve analysis of 15 differentially expressed miRNAs of gastric cancer tissues vs. adjacent non-tumor tissues in the analysis set. miRNAs upregulated in gastric cancer were **a** miR-23a, **b** miR-34c, **c** miR-423, **d** miR-496, **e** miR-500b, **f** miR-542, **g** miR-1228, and **h** miR-3687. miRNAs downregulated in gastric cancer were **i** miR-30a, **j** miR-133a-1, **k** miR-133a-2, **l** miR-133b, **m** miR-139, **n** miR-143, and **o** miR-195. The area under the receiver operating characteristic curve values were calculated to estimate the diagnostic performance of the differentially expressed miRNAs in gastric cancer. Data are expressed as the means ± standard deviations (SDs). *P < 0.05, **P < 0.01, ***P < 0.001

**Fig. 3:**
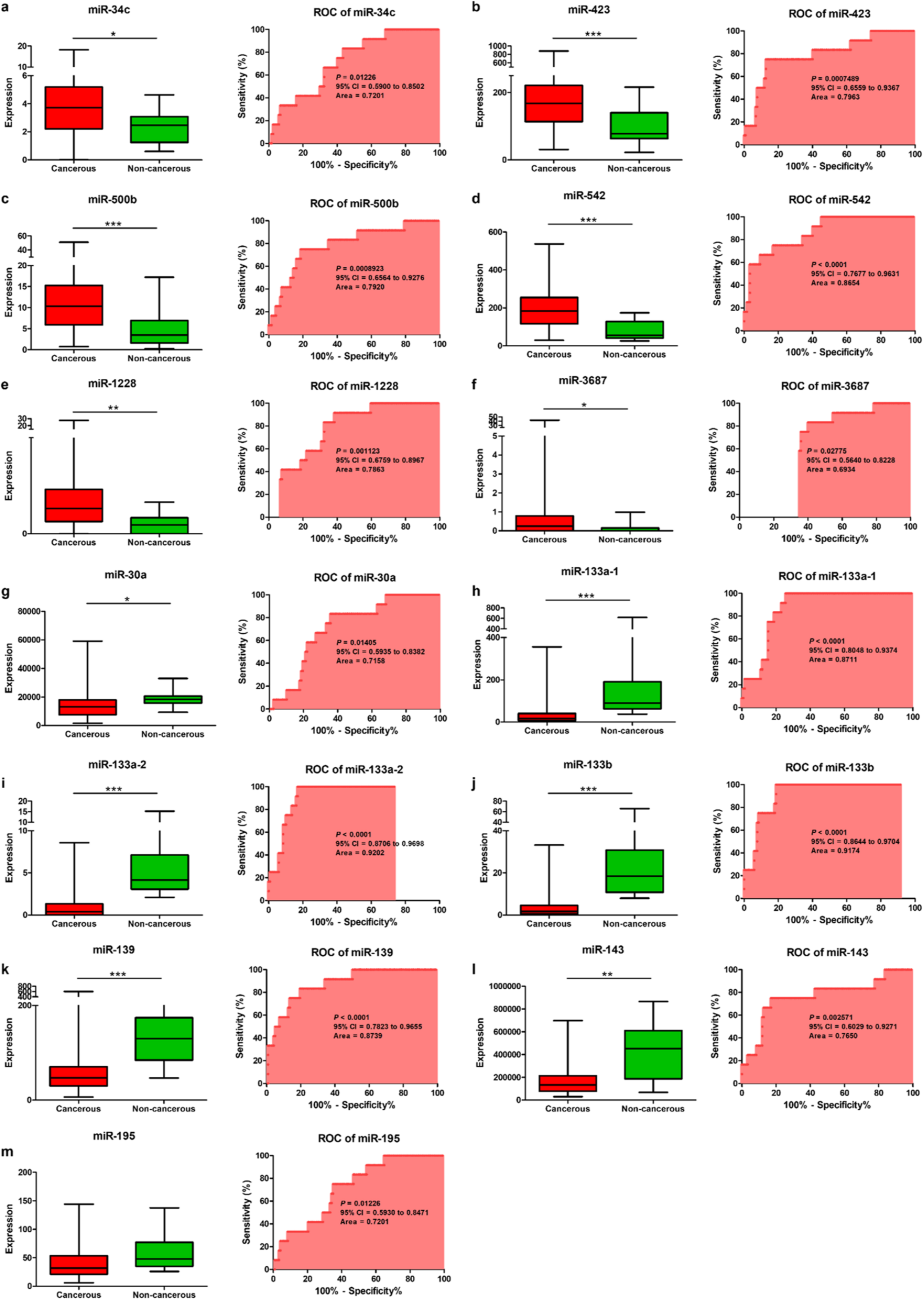
Expression levels of 13 differentially expressed miRNAs and receiver operating characteristic curve analysis of 13 differentially expressed miRNAs of gastric cancer tissues vs. adjacent non-tumor tissues in the validation set. miRNAs upregulated in gastric cancer were **a** miR-34c, **b** miR-423, **c** miR-500b, **d** miR-542, **e.** miR-1228, and **f.** miR-3687. miRNAs downregulated in gastric cancer were **g** miR-30a, **h** miR-133a-1, **i** miR-133a-2, **j** miR-133b, **k** miR-139, **l** miR-143, and **m** miR-195. The area under the receiver operating characteristic curve values were calculated to estimate the diagnostic performance of the differentially expressed miRNAs in gastric cancer. Data are expressed as the means ± standard deviations (SDs). *P < 0.05, **P < 0.01, ***P < 0.001

The TCGA data was split in two separate sets: the analysis set, and the validation set. For the analysis and validation of selecting potential diagnosis biomarker for gastric cancer using the area under the curve (AUC) analysis, the analysis set was created using 70% of total TCGA data. The remaining 30%, was used to the validation set [26]. In the analysis set, we determined the diagnostic usefulness of the 15 DE miRNAs as potential GC biomarkers using receiver operating characteristic (ROC) curve analysis. The AUC of all these 15 DE miRNAs in GC was more than 0.8. To explore the diagnostic usefulness of the 15 candidate miRNAs, we assessed their expression levels and discovered that they were considerably higher or lower in GC tissues than in adjacent non-tumor tissues (Fig. 2a–o). Then, the diagnostic value of 13 DE miRNAs was confirmed using ROC curve analysis again in the validation set. As a result of ROC analysis in the validation set, the diagnostic value of 13 DE miRNAs was confirmed, except for 2 DE miRNAs with *p*-values not less than 0.05 (Fig. 3a–m).

### Analysis of high-throughput RNA sequencing data

**Fig. 4:**
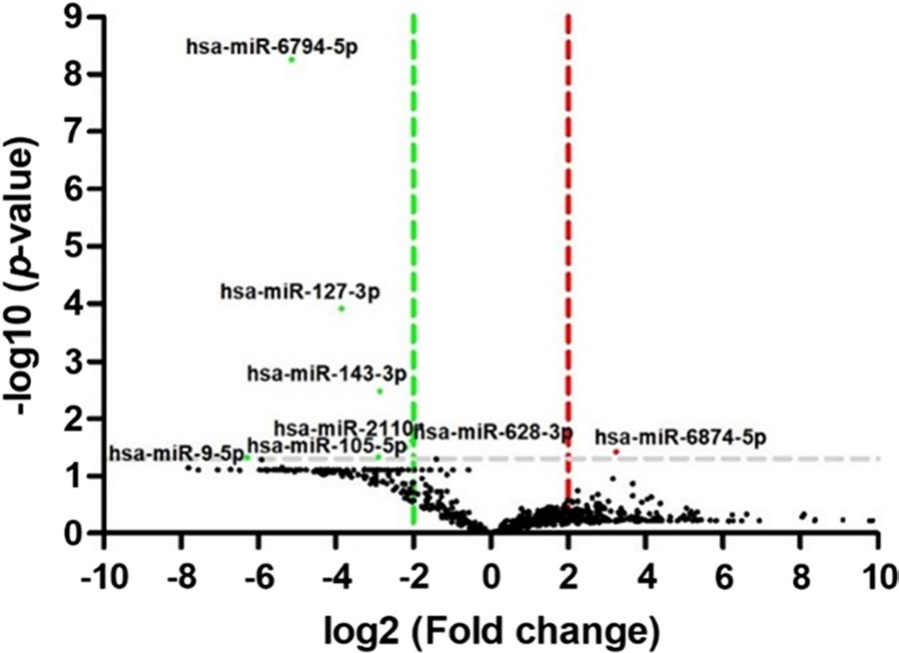
Volcano plot of the differential expression analysis of miRNAs. The expression of miRNAs in the gastric cancer plasma was compared to that in the non-tumor plasma. Log2 fold changes are plotted on the *x*-axis, and the – log10 of the *p*-value is plotted on the *y*-axis. Red: upregulated miRNA; Green: downregulated miRNA

The expression levels of miRNAs in six GC patients and two healthy subjects were identified using high-throughput RNA sequencing, which was commercially performed by ebiogen, Inc. (ebiogen, Seoul, South Korea). Subsequently, we analyzed the results of the DE miRNAs using ExDEGA GraphicPlus 2.0 (ebiogen, Seoul, South Korea). The DE miRNAs were selected using a *p*-value < 0.05 and cut-off of fold changes > 2. A volcano plot of the DE miRNAs was generated with GraphPad Prism 6.0 (Fig. 4). The upregulated DE miRNA was hsa-miR-6874-5p. The seven downregulated DE miRNAs were hsa-miR-9-5p, hsa-miR-105-5p, hsa-miR-127-3p, hsa-miR-143-3p, hsa-miR-628-3p, hsa-miR-2110, and hsa-miR-6794-5p (Table 4).

**Table 4:** The up- and downregulated differentially expressed miRNAs in patients with gastric cancer

DE miRNA	Accession	Fold change	*p*-value
*Upregulated*			
hsa-miR-6874-5p	MI0022721	3.235	0.038
*Downregulated*			
hsa-miR-9-5p	MI0000466	− 6.265	0.048
hsa-miR-105-5p	MI0000111	− 2.900	0.047
hsa-miR-127-3p	MI0000472	− 3.857	< 0.001
hsa-miR-143-3p	MI0000459	− 2.857	0.003
hsa-miR-628-3p	MI0003642	− 2.023	0.025
hsa-miR-2110	MI0010629	− 2.191	0.032
hsa-miR-6794-5p	MI0022639	− 5.158	< 0.001

*DE* differentially expressed; fold change > 2

### Selection of miR-143-3p as a diagnostic biomarker in gastric cancer

**Fig. 5:**
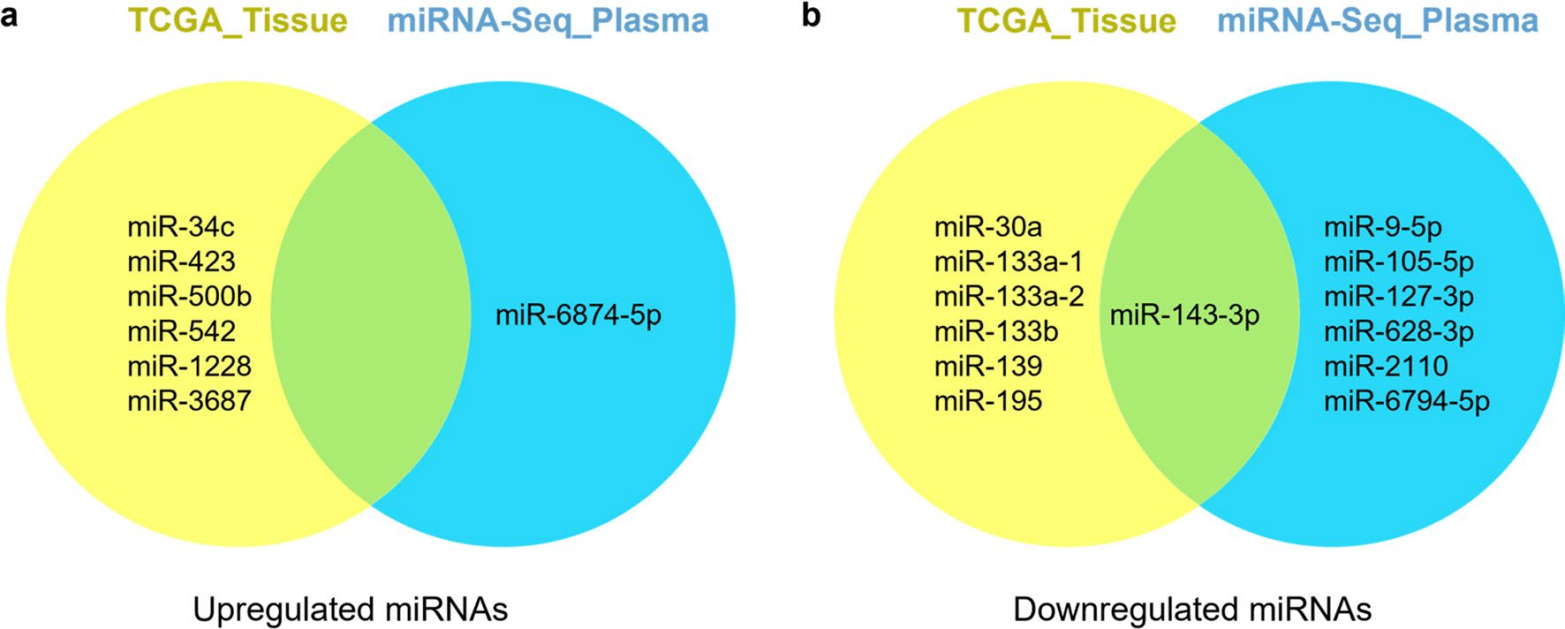
Venn diagram of differentially expressed miRNAs in gastric cancer. **a.** Upregulated miRNAs. **b.** Downregulated miRNAs

Based on the TCGA data, the upregulated miRNAs were miR-34c, miR-423, miR-500b, miR-542, miR-592, miR-652, and miR-1228. The expression levels of these miRNAs were higher in GC tissues than adjacent non-tumor tissues. On the other hand, only miR-6874-5p was upregulated in the miRNA expression profile for GC plasma. miR-143-3p was downregulated in according to both the TCGA data and the miRNA expression profile (Fig. 5a, b). Therefore, we selected miR-143-3p as a suitable diagnostic biomarker for GC patients.

### Validation of miR-143-3p expression level by quantitative real-time PCR

**Fig. 6:**
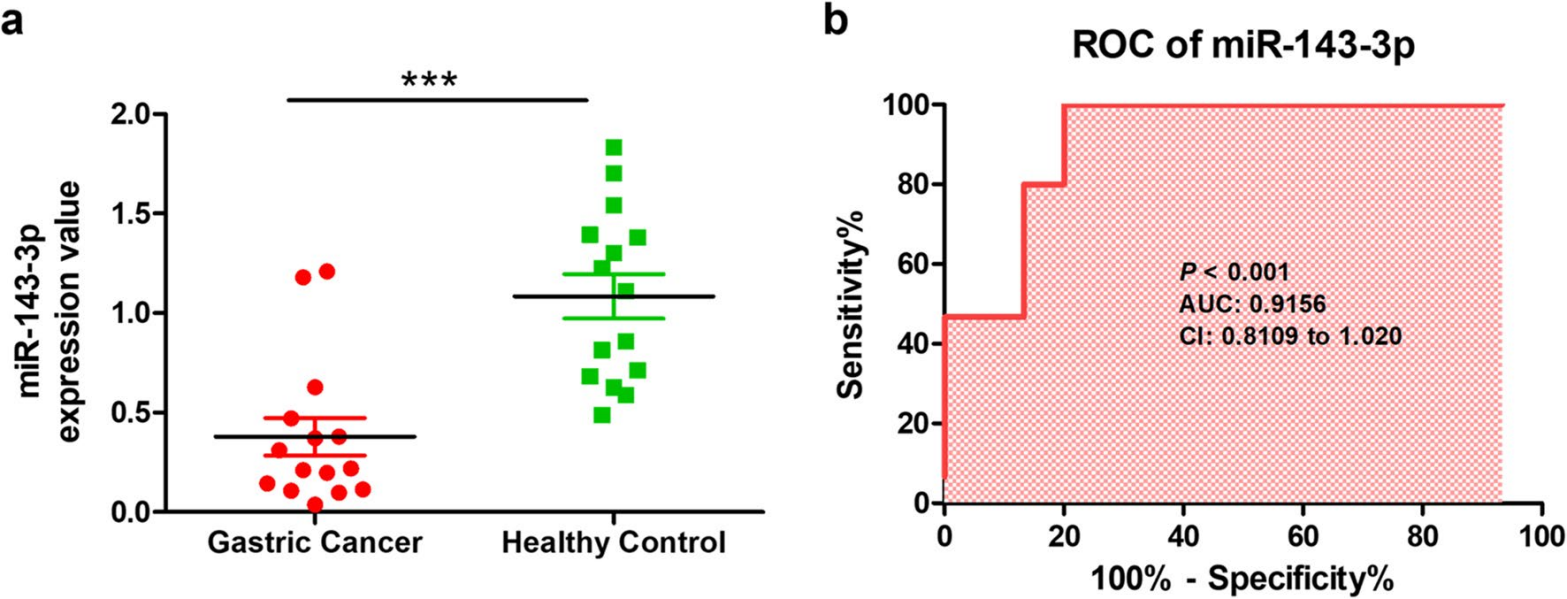
The clinical value of miR-143-3p in plasma of patients with gastric cancer. **a** The expression levels of miR-143-3p in plasma of patients with gastric cancer. **b** The results of receiver operating characteristic curve analysis to distinguish healthy controls and patients with gastric cancer. ***P < 0.001

Quantitative real-time PCR analysis is a widely used method for validation for confirming gene expression results from RNA sequencing analysis [27]. We validated the expression level of miR-143-3p using qPCR. As shown in Fig. 6a, the expression levels of miR-143-3p were significantly downregulated in gastric cancer patients compared with the healthy subjects. The diagnostic performance of miR-143-3p were determined by ROC curve analysis. The AUC of miR-143-3p was 0.9156 (95% CI = 0.8109 to 1.020, *P* < 0.001) (Fig. 6b).

### Target prediction analysis for miR-143-3p

**Fig. 7:**
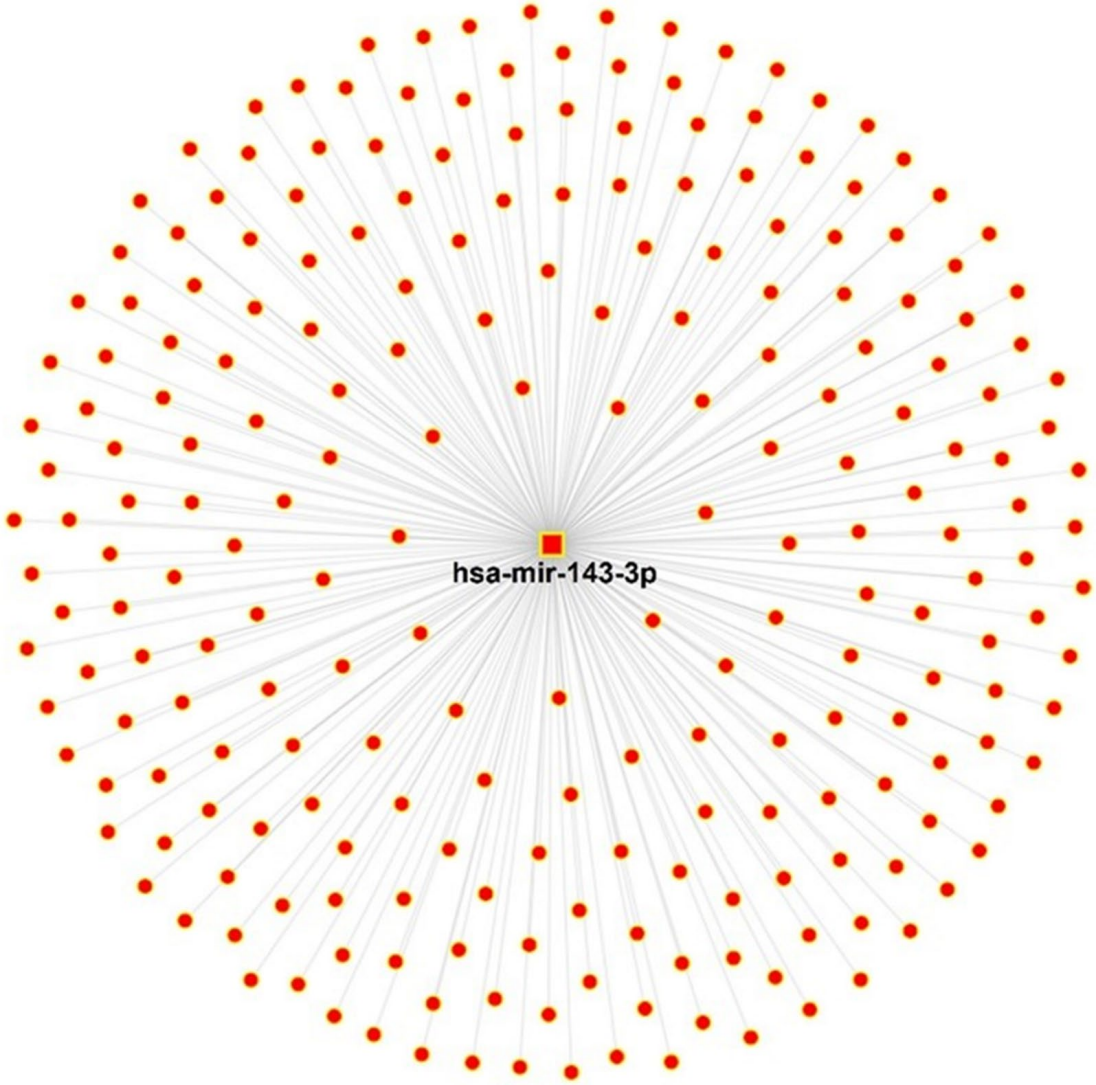
Interaction network of hsa-miR-143-3p and potential target genes

To precisely analyze the functional characterization of hsa-miR-143-3p in GC, potential target mRNAs were predicted. By constructing a miRNA-mRNA interaction network, 228 potential target genes of miR-143-3p were gained (Fig. 7). The predicted target genes of hsa-miR-143-3p are reported in Additional file 1: Table S2.

### Gene ontology and KEGG analysis

**Fig. 8:**
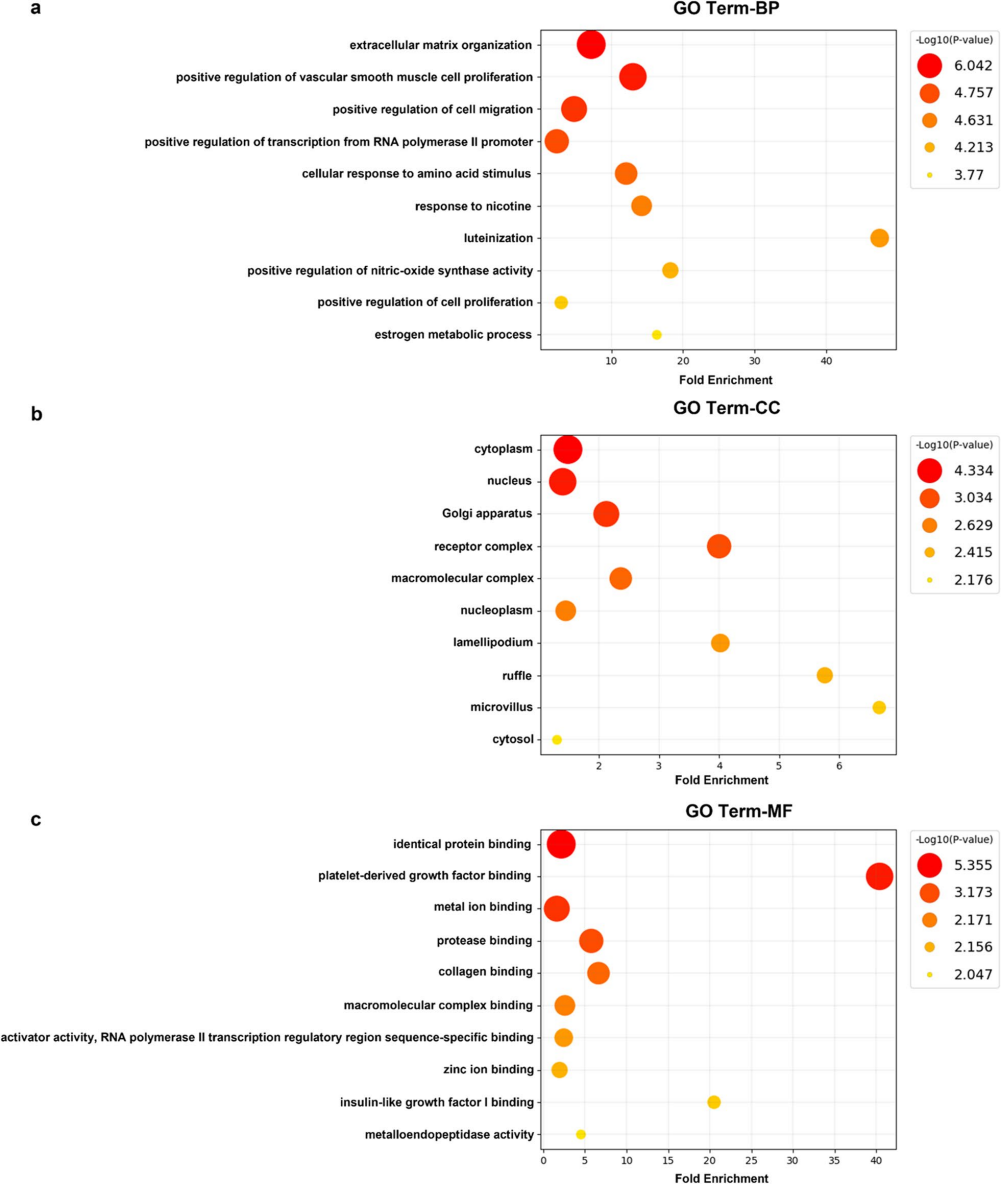
Gene ontology analysis of potential target genes. **a** Categories of target genes in biological processes. **b** Categories of target genes in cellular components. **c** Categories of target genes in molecular functions. The top 10 significantly affected GO terms (– log10 (*p*-value)) are listed along the *y*-axes, while the *x*-axes denote fold enrichment

**Fig. 9:**
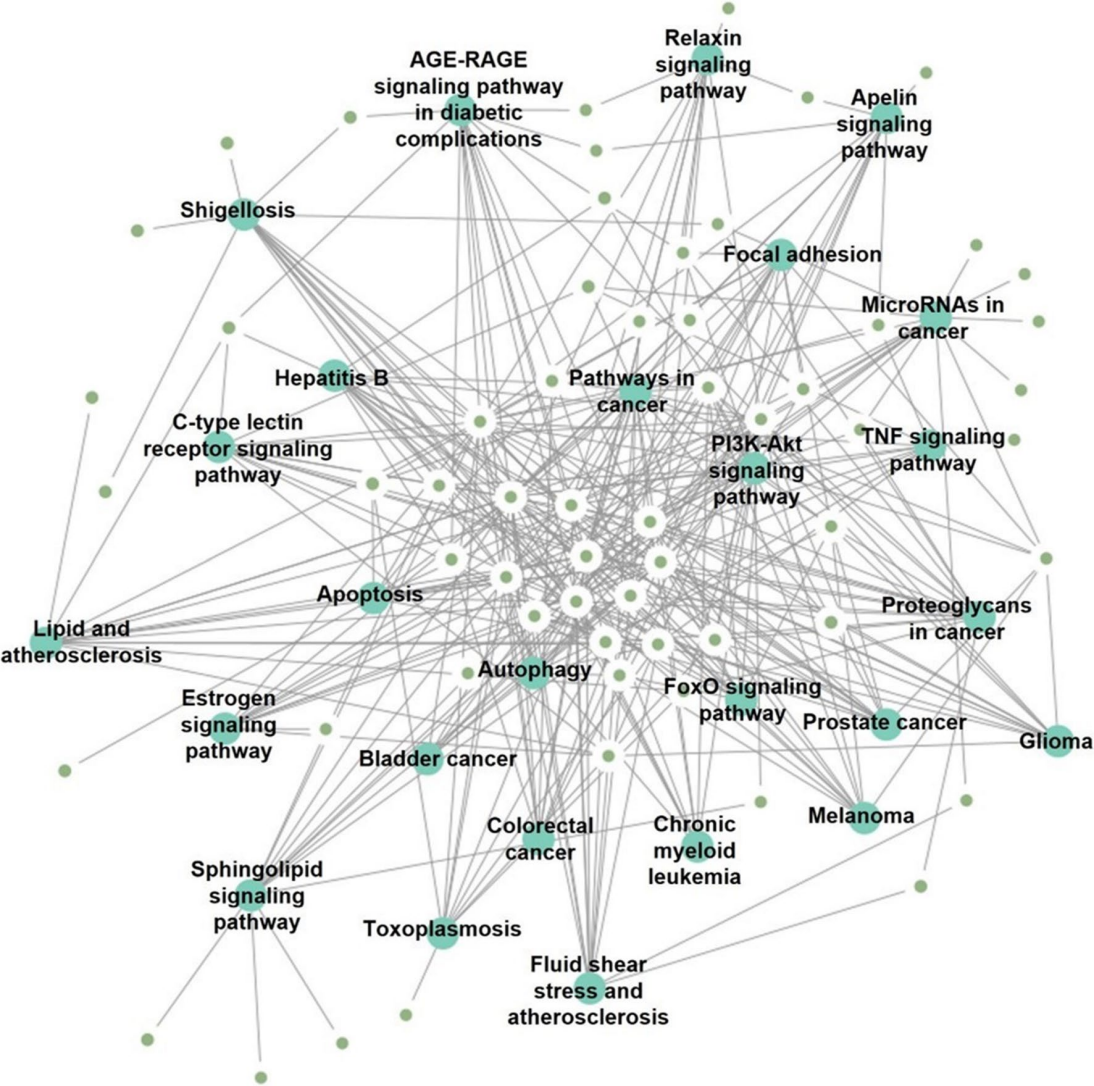
Pathway analysis of potential target genes. Enriched pathway interaction network identified by the Kyoto Encyclopedia of Genes and Genomes (KEGG) database

The 228 target genes of hsa-miR-143-3p were used to perform the GO and KEGG enrichment analysis. As a result of the GO analysis, we obtained a list of functional GO terms in three categories: BP, CC, and MF. The BP category contains the essential GO terms extracellular matrix organization (GO:0030198), positive regulation of vascular smooth muscle cell proliferation (GO:1904707), and positive regulation of cell migration (GO:0030335) (Fig. 8a). The CC category relates to the cytoplasm (GO:0005737), nucleus (GO:0005634), and Golgi apparatus (GO:0005794) (Fig. 8b). The MF category includes identical protein binding (GO:0042802), platelet-derived growth factor binding (GO:0048407), and metal ion binding (GO:0046872) (Fig. 8c). KEGG pathways indicate the functional pathways that contribute to the processes of GC [28–30]. The pathway enrichment analysis of target genes indicated pathways in cancer (KEGG:05200), phosphoinositide 3-kinase (PI3K)-protein kinase B (Akt) signaling (KEGG:04151), proteoglycans in cancer (KEGG:05205), and microRNAs in cancer (KEGG:05206) (Fig. 9 and Table 5). The KEGG pathway related target genes are shown in Additional file 1: Table S3.

**Table 5:** KEGG pathway enrichment analysis of the selected target genes

KEGG ID	KEGG term	Gene count	*p*-value
KEGG:05200	Pathways in cancer	24	< 0.001
KEGG:04151	PI3K–Akt signaling pathway	19	< 0.001
KEGG:05205	Proteoglycans in cancer	18	< 0.001
KEGG:05206	MicroRNAs in cancer	17	< 0.001
KEGG:04933	AGE–RAGE signaling pathway in diabetic complications	15	< 0.001
KEGG:05131	Shigellosis	15	< 0.001
KEGG:05161	Hepatitis B	15	< 0.001
KEGG:04510	Focal adhesion	14	< 0.001
KEGG:05417	Lipid and atherosclerosis	14	< 0.001
KEGG:04071	Sphingolipid signaling pathway	13	< 0.001
KEGG:05215	Prostate cancer	13	< 0.001
KEGG:04371	Apelin signaling pathway	12	< 0.001
KEGG:04625	C-type lectin receptor signaling pathway	12	< 0.001
KEGG:04926	Relaxin signaling pathway	12	< 0.001
KEGG:05214	Glioma	12	< 0.001
KEGG:05418	Fluid shear stress and atherosclerosis	12	< 0.001
KEGG:04068	FoxO signaling pathway	11	< 0.001
KEGG:04140	Autophagy	11	< 0.001
KEGG:04210	Apoptosis	11	< 0.001
KEGG:04915	Estrogen signaling pathway	11	< 0.001
KEGG:05218	Melanoma	11	< 0.001
KEGG:04668	TNF signaling pathway	10	< 0.001
KEGG:05145	Toxoplasmosis	10	< 0.001
KEGG:05210	Colorectal cancer	10	< 0.001
KEGG:05220	Chronic myeloid leukemia	9	< 0.001
KEGG:05219	Bladder cancer	7	< 0.001

*KEGG* Kyoto Encyclopedia of Genes and Genomes

### Protein–protein interaction network analysis

**Fig. 10:**
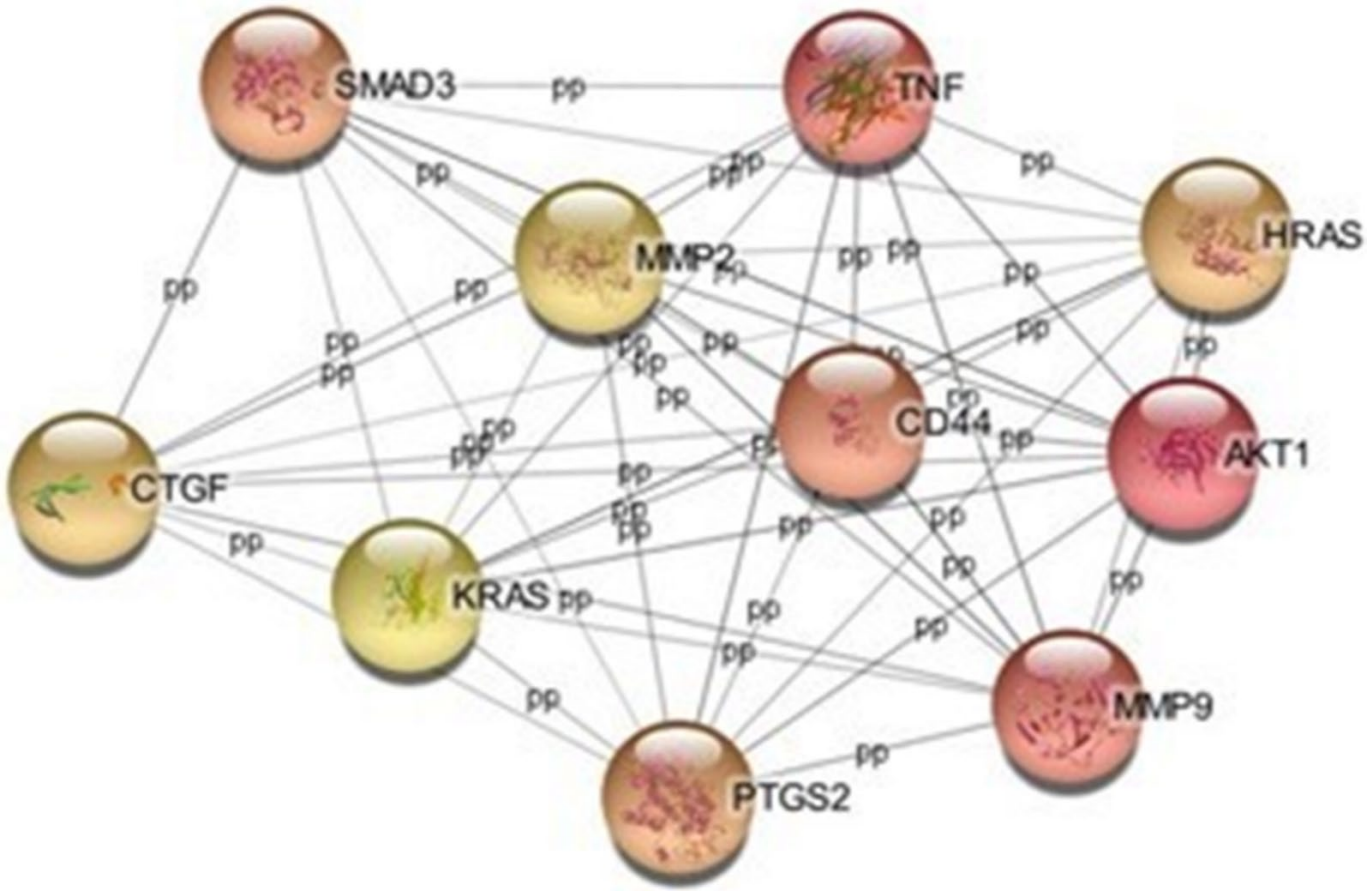
Protein–protein interaction networks of the top 10 hub genes

To explore the hub genes of the DE miRNAs, the PPI network was analyzed with the Maximal Clique Centrality (MCC) algorithm to identify the top 10 hub genes [22, 31]: matrix metallopeptidase 2 (MMP2), CD44 molecule (CD44), SMAD family member 3 (SMAD3), connective tissue growth factor (CTGF), KRAS proto-oncogene (KRAS), prostaglandin-endoperoxide synthase 2 (PTGS2), matrix metallopeptidase 9 (MMP9), AKT serine/threonine kinase 1 (AKT1), HRas proto-oncogene (HRAS), and tumor necrosis factor (TNF) (Fig. 10).

## Discussion

The lack of markers for detection limits the prevention and treatment of GC, leading to a low survival rate for patients [32]. Although there is very wide global variation, the overall five-year survival rate of patients with GC is approximately 20–30% in most countries [33]. The blocking of GC can reduce the side effects of unnecessary palliative radiotherapy and chemotherapy and increase the survival rate of GC patients. To identify GC, we must explore the potential biomarkers useful for its diagnosis of GC. The miRNA expression in GC tissue samples may have an important role for miRNAs in tumorigenesis and progression of the disease and differentially expressed miRNAs correlate with the pathological type of GC [34, 35]. miRNA in plasma could serve as potential biomarker in detection of GC [34, 36]. Several studies discovered the identical trend of alteration between tissue miRNAs and plasma miRNAs [37, 38]. This suggests that plasma biomarkers may be an alternative to tissue biomarkers. Thus, it would be more clinical significance, if we can discern the miRNAs that are common differentially expressed in GC tissue and plasma.

Shin et al. [39], proposed three miRNA signatures (miR-627, miR-629, and miR-652) as potential diagnostic markers of GC. Li et al. [40], revealed that miR-199a-3p in plasma may be considered as a potential diagnostic biomarker for GC detection. In this study, we analyzed sequencing data for miRNAs in 389 GC samples and 41 adjacent non-tumor tissues from TCGA. Altered miRNA expression patterns were identified between GC and non-tumor tissues, implying a potential diagnostic role for miRNAs in GC. As a result of the hierarchical clustering analysis, 111 DE miRNAs were identified. Furthermore, 13 DE miRNAs of diagnostic value were selected by performing ROC analysis. Among the 13 DE miRNAs, we identified that the expression of miR-143-3p was significantly downregulated in GC plasma compared to healthy subjects by high-throughput small RNA profiling. miR-143-3p is well-conserved in vertebrates and is known to be a potential regulator of tumor growth [41, 42]. Additionally, miR-143-3p has been reported to inhibit the development of ovarian and breast cancer [42, 43]. miR-143-3p may serve as a diagnostic marker of osteosarcoma, chondrosarcoma, and bladder cancer [44–46]. miR-143-3p is downregulated in GC and has tumor suppression [47]. CircFOXO3 targeting miR-143-3p promotes GC cell proliferation and migration by upregulating USP44 expression [48]. Circ_0006089, a circular RNA, regulates the miR-143-3p/PTBP3 axis and PI3K/AKT signaling pathway to promote the progression of GC [49]. Therefore, miR-143-3p may affect GC progress. Furthermore, evaluating the diagnostic accuracy of miRNA based on GC tissue confirmed that miR-143-3p has a high diagnostic value as a single molecule [35]. Therefore, based on previous studies and our findings, we have demonstrated that miR-143-3p may be valuable as a diagnostic biomarker for GC. When we validated the diagnostic value with 21 GC and 17 healthy controls, the level of miR-143-3p was significantly downregulated in GC patients. This finding needs to validate more with large numbers of patients.

In the present study, we identified a stronger molecular biomarker for GC by targeting miRNAs identically downregulated in two profile datasets. The miRNA–mRNA interaction network was constructed, resulting in the identification of miR-143-3p related 228 target genes. To investigate their functional levels, we analyzed the GO functions and KEGG pathways. A PPI network was constructed to identify the top 10 hub genes. SMAD3, TNF, MMP2, MMP9, CTGF, CD44 and AKT1 are associated with atherosclerosis [50–56]. Gastric cancer and atherosclerosis are known to share many etiological and mechanistical processes, as well as several important molecular pathways [57]. The GO analysis revealed that biological processes were significantly enriched for GC development. The extracellular matrix (ECM) is an interconnected macromolecular structure that contributes to cell migration and cancer development [58, 59]. Moreira et al., described how the disruption of the ECM organization impairs GC function and tissue structure, eventually leading to the progression of gastric cancer [60]. Through KEGG analysis, we identified pathways significant in the pathogenesis of GC, such as pathways in cancer, PI3K–Akt signaling, proteoglycans in cancer, and microRNAs in cancer. PI3K–Akt signaling may play a significant role in potential therapeutic targets for gastric cancer [61, 62]. Ye et al., proved that inhibiting PI3K signaling reduced the expression of p-AKT and MMP2, thereby suppressing the proliferative activities and metastatic capabilities of gastric cancer cells [63]. Our study shows crucial molecular pathways and provides insights into potential targets for GC. The results of our analyses are greatly significant for the investigation of the role of miR-143-3p and related target genes in the progression of GC. However, the conclusions of our study need to be further evaluated with a larger dataset.

Collectively, our study suggests that hsa-miR-143-3p represents a potential biomarker for the diagnosis of GC, with the potential to play a significant role through the pathways involved in GC progression. Nevertheless, its clinical application warrants further research.

## Supplementary Information

**Additional file 1**. **Table S1**: The list of 111 DE miRNAs. **Table S2**: The list of predicted target genes. **Table S3**: The KEGG pathways related target genes.

## Data Availability

The datasets generated and/or analysed during the current study are available in the Figshare, [https://figshare.com/articles/dataset/The_miRNA_expression_profile_xlsx/21959984]. The transcription sequencing data and corresponding clinical information of TCGA-gastric cancer cohort are available from TCGA (https://portal.gdc.cancer.gov/). Additional data can be requested from the corresponding author.

## Abbreviations

GC: Gastric cancer
miRNA: MicroRNA
TCGA: The cancer genome atlas
qPCR: Quantitative real-time PCR
GO: Gene ontology
KEGG: Kyoto Encyclopedia of Genes and Genomes
PPIs: Protein–protein interactions
ROC: Receiver operating characteristic curve
AUC: Area under the curve
MCC: Maximal clique centrality
